# Urine autotaxin levels reflect the disease activity of sarcoidosis

**DOI:** 10.1038/s41598-022-08388-6

**Published:** 2022-03-14

**Authors:** Koji Murakami, Tsutomu Tamada, Daisuke Saigusa, Eisaku Miyauchi, Masayuki Nara, Masakazu Ichinose, Makoto Kurano, Yutaka Yatomi, Hisatoshi Sugiura

**Affiliations:** 1 Department of Respiratory Medicine, Tohoku University Graduate School of Medicine, 1-1 Seiryo-machi, Aoba-ku, Sendai, 980-8574 Japan; 2 Laboratory of Biomedical and Analytical Sciences, Faculty of Pharma-Science, Teikyo University, Tokyo, Japan; 3 National Hospital Organization Akita National Hospital, Yurihonjo, Japan; 4 Osaki Citizen Hospital, Osaki, Japan; 5 Department of Clinical Laboratory, Tokyo University Graduate School of Medicine, Tokyo, Japan

**Keywords:** Biochemistry, Biomarkers, Diseases

## Abstract

Since the clinical outcome of patients with sarcoidosis is still unpredictable, a good prognostic biomarker is necessary. Autotaxin (ATX) and phosphatidylserine-specific phospholipase A1 (PS-PLA1) function as main enzymes to produce lysophospholipids (LPLs), and these enzymes are attracting attention as useful biomarkers for several chronic inflammatory diseases. Here, we investigated the relationships between LPLs-producing enzymes and the disease activity of sarcoidosis. In total, 157 patients with sarcoidosis (active state, 51%) were consecutively enrolled. Using plasma or urine specimens, we measured the values of LPLs-producing enzymes. Urine ATX (U-ATX) levels were significantly lower in the active state compared to those in the inactive state, while the plasma ATX (P-ATX) and PS-PLA1 levels showed no significant difference between these two states. Concerning the comparison with existing clinical biomarkers for sarcoidosis, U-ATX showed a weak negative correlation to ACE, P-ATX a weak positive correlation to both ACE and sIL-2R, and PS-PLA1 a weak positive one to sIL-2R. Notably, only the U-ATX levels inversely fluctuated depending on the status of disease activity whether OCS had been used or not. These findings suggest that U-ATX is likely to be a novel and useful molecule for assessing the disease activity of sarcoidosis.

## Introduction

Sarcoidosis is a systemic granulomatous disorder of unknown etiology. The clinical features of sarcoidosis are quite different among regions, races and ethnicities and the outcomes of this disease also greatly differ^1–4^. Because of the diversity in the pathogenesis of sarcoidosis, it is difficult to predict the clinical outcome of sarcoidosis, ranging from spontaneous remission to severe dysfunction of major organs^3,5^. Increased levels in several serum laboratory tests such as angiotensin converting enzyme (ACE), soluble interleukin-2 receptor (sIL-2R) and lysozyme were observed at the time of diagnosis. These molecules seem somewhat useful for monitoring the disease activity, but not enough to estimate the changes in severity or to predict the prognosis of sarcoidosis. Concerning the treatment for patients with severe sarcoidosis or severe dysfunction of major organs, oral corticosteroids (OCS) are commonly used as a first line therapy, but long-term use of OCS may cause many serious adverse effects including hyperglycemia, immunodeficiency, osteoporosis and so on. If we can find useful monitoring or prognostic biomarkers associated with the pathophysiology of sarcoidosis, it would be helpful not only for improving the management of patients with sarcoidosis, but also for developing novel treatment strategies.

Eicosanoid mediators such as prostaglandins and leukotrienes have long been studied as first-generation bioactive lipids. On the other hand, lysophospholipids (LPLs), such as lysophosphatidic acid (LPA), sphingosine 1-phosphate (S1P), and lysophosphatidylserine (LysoPS), have emerged and been attracting attention to researchers as second-generation bioactive lipids in recent decades^6–9^. Among these LPLs, LPA and LysoPS are produced extracellularly, whereas S1P is produced intracellularly and released extracellularly. Relatively high concentrations of LPLs are known to exist in body fluids such as blood (plasma, serum) and function through specific G protein-coupled receptors expressed on the surfaces of target cells. Although assays of these lipids themselves are possible, the regulation of the metabolism of LPL mediators is complicated and it is not LPLs themselves but the enzymes catalyzing their production, that are encoded by the genomic DNA. Therefore, LPLs-producing enzymes such as autotaxin (ATX) or phosphatidylserine-specific phospholipase A1 (PS-PLA1) and even a carrier such as apolipoprotein M (ApoM) are more promising as alternate biomarkers for LPA, LysoPS and S1P, respectively^6,7,10–12^. ATX is a secreted lysophospholipase D catalyzing the extracellular hydrolyses of lysophosphatidylcholine to LPA and is considered to be responsible for the synthesis of the majority of extracellular LPA^13^. ATX/LPA signaling plays important roles in the pathophysiology of several chronic inflammatory diseases such as interstitial lung diseases, cancer, liver cirrhosis and atherosclerosis^14–18^. It has been reported that ATX can bind to lymphocytes, and LPA or ATX/LPC strongly enhances the trans-endothelial migration of integrin-arrested T cells across an endothelial monolayer and promotes lymphocytes to enter lymphoid organs from the blood^19,20^. In mouse models with rheumatoid arthritis, ATX proteins are expressed on synovial fibroblasts and its expression is markedly stimulated by TNF, which is one of the crucial mediators for granuloma formation^21^. PS-PLA1 is one of the enzymes that is responsible for LysoPS production. Unlike the ATX/LPA axis, the biological roles of PS-PLA1/LysoPS in the pathogenesis of those diseases have not been well studied. Recent studies suggest that the PS-PLA1/LysoPS axis is partially involved in the pathogenesis of cancer, acute coronary syndrome, and hyperthyroidism^22–25^. However, although it has been reported that the PS-PLA1/LysoPS axis inhibits mitogen-induced T-cell activation in vitro^26^, the association between the LPLs-producing enzymes/LPLs signaling and the disease activity of sarcoidosis remains to be elucidated.

To investigate potential diagnostic of prognostic biomarkers for sarcoidosis, we collected over 150 plasma and urine samples from patient with active or inactive sarcoidosis and analyzed the relationships between LPLs-producing enzymes and the disease activity. Because the present study aims to find novel clinically useful biomarkers and apply them to the daily examination in a clinical setting, we focused on the values of ATX and PS-PLA1, both of which are present in body fluids and have been used as an alternative for the value of LPA and LysoPS, respectively^27^.

## Results

### Clinical characteristics of subjects

During the inclusion period, 53 out of 211 patients with sarcoidosis were excluded because of ongoing OCS therapy at the time of enrollment and 1 patient because of chronic hepatitis, and then 157 patients were finally enrolled in this study. Because the plasma- ATX (P-ATX) levels are known to differ between genders^28^, we first analyzed the data among gender groups in addition to the whole subjects group. The clinical characteristics of subjects are summarized in Table 1. A subtotal of 100 (64%) patients were female. Female subjects were more likely to be older, and showed a lower smoking index and higher prevalence of Stage 1 lung lesions. The enrolled subjects had almost similar characteristics with a population in a previous sarcoidosis cohort study in Japan^29,30^. The disease activity was not significantly different between male and female groups.

**Table 1:** Characteristics of patients with sarcoidosis.

	All	Male	Female	*p* value
Number, n (%)	157	57(36%)	100 (64%)	
Age (years)	52 (37–61)	46 (34–61)	58 (46–66)	0.001*
BMI (kg/cm^2^)	22.2 (20.2–24.2)	22.6 (20.5–25.3)	22.0 (19.9–23.9)	0.08
Smoking index (Pack-years)	1 (0–11)	9 (5–21)	0 (0–6)	< 0.001*
**Sarcoidosis**				
Stage 0, n (%)	4 (3%)	2 (4%)	2 (2%)	0.62
Stage 1, n (%)	76 (48%)	21 (37%)	55 (55%)	0.03*
Stage 2, n (%)	68 (43%)	29 (50%)	39 (39%)	0.15
Stage 3 and 4, n (%)	9 (6%)	5 (9%)	4 (4%)	0.29
**Organ involvement**				
Eyes, n (%)	87 (55%)	27 (47%)	60 (60%)	0.13
Skin, n (%)	40 (25%)	10 (18%)	30 (30%)	0.09
Heart, n (%)	19 (12%)	6 (11%)	13 (13%)	0.61
Others, n (%)	53 (34%)	16 (28%)	37 (37%)	0.26
Activity (Active state), n (%)	80 (51%)	25 (44%)	55 (55%)	0.18
ACE (U/L)	17.3 (13.5–22.8)	16.1 (12.9–21.8)	18.5 (14.1–23.5)	*0.24*
sIL-2R (U/mL)	525 (353–843)	550 (310–915)	518 (368–823)	*0.75*
KL-6 (U/mL)	353 (253–530)	435 (245–613)	335 (253–436)	0.11
%VC	106 (96–114)	106 (92–113)	105 (96–116)	0.35
P-ATX (mg/dL)	0.94 (0.78–1.12)	0.79 (0.71–0.92)	1.02 (0.89–1.20)	< 0.001*
U-ATX ($\upmu$ g/gCre)	4.34 (1.99–7.18)	3.69 (1.78–5.31)	5.35 (2.23–8.76)	0.01*
PS-PLA1 (ng/mL)	15.5 (12.9–18.3)	16.5 (14.6–18.6)	14.8 (12.0–17.6)	0.001*
eGFR (mL/min)	78 (64–89)	79 (65–93)	76 (63–87)	0.21
Oral Corticosteroid	None	None	None	

Data are expressed as median and interquartile range (IQR, Q1-Q3). The clinical characteristics were compared using Mann–Whitney U-test or Fisher’s exact test. Statistical significance was accepted as *p* < 0.05 and marked by an asterisk.

The subjects were classified into two groups according to their disease activity, that is, 80 patients in an active state and 77 in an inactive state (Table 2). Subjects in an active state showed a lower body-mass index (BMI), a higher prevalence of eyes and cardiac involvement and higher values in ACE, sIL-2R and KL-6.

**Table 2:** Comparison of characteristics of the patients with sarcoidosis between active and inactive state.

		All		Male			Female		
	Active	Inactive	*p* value	Active	Inactive	*p* value	Active	Inactive	*p* value
Number	80	77		25	32		55	45	
Gender (Female) n (%)	55 (69%)	45 (58%)	0.18						
Age (years)	54 (43–65)	56 (39–66)	0.73	43 (27–65)	48 (36–58)	0.53	56 (46–64)	59 (41–70)	0.29
BMI (kg/cm^2^)	21.5 (19.9–23.8)	23.1 (20.7–24.4)	0.02*	21.5 (19.9–24.1)	23.6 (21.6–25.9)	0.02*	21.5 (19.9–23.8)	22.5 (20.0–24.1)	0.33
Smoking (Pack-years)	0 (0–12)	3 (0–10)	0.26	9 (3–33)	8 (6–18)	0.74	0 (0–0)	0 (0–7)	0.14
**Sarcoidosis**									
Stage0, n (%)	1 (1%)	3 (4%)	0.36	1 (4%)	1 (3%)	1.00	0 (0%)	2 (4%)	0.20
Stage1, n (%)	37 (46%)	39 (50%)	0.48	8 (32%)	13 (41%)	0.50	29 (53%)	26 (58%)	0.61
Stage2, n (%)	39 (49%)	29 (38%)	0.08	14 (56%)	15 (47%)	0.49	25 (45%)	14 (31%)	0.10
Stage3&4, n (%)	3 (4%)	6 (8%)	0.32	2 (8%)	3 (9%)	1.00	1 (2%)	3 (7%)	0.32
**Organ involvement**									
Eye, n (%)	51 (64%)	36 (47%)	0.03*	14 (56%)	13 (41%)	0.25	37 (67%)	23 (51%)	0.10
Skin, n (%)	23 (29%)	17 (22%)	0.34	4 (16%)	6 (19%)	0.79	19 (35%)	11 (24%)	0.27
Heart, n (%)	15 (19%)	4 (5%)	0.01*	4 (16%)	2 (6%)	0.23	11 (20%)	2 (4%)	0.03*
Others, n (%)	33 (41%)	20 (26%)	0.20	9 (35%)	7 (22%)	0.018*	24 (44%)	13 (29%)	0.13
ACE (U/L)	19.8 (15.4–26.7)	16.1 (12.1–19.3)	< 0.0001*	18.0 (13.7–26.0)	15.5 (12.6–20.6)	0.09	20.9 (15.6–27.4)	16.1 (11.7–18.9)	< 0.0001*
sIL-2R (U/mL)	700 (447–1100)	419 (303–588)	< 0.0001*	846 (542–1387)	392 (208–479)	0.0002*	691 (417–1040)	424 (343–573)	0.0007*
KL-6 (U/mL)	409 (278–551)	302 (228–394)	0.01*	541 (375–695)	302 (208–479)	0.07	362 (263–485)	294 (243–396)	0.16
%VC	105 (96–114)	106 (96–114)	0.61	104 (68–113)	106 (92–113)	0.56	105 (96–114)	107 (96–116)	0.68
eGFR (mL/min)	77 (63–88)	79 (66–89)	0.37	77 (64–102)	81 (68–90)	0.43	75 (61–87)	77 (64–88)	0.73
Oral Corticosteroid	None	None	None	None	None		None	None	

Data are expressed as median and interquartile range (IQR, Q1-Q3). The clinical characteristics were compared using Mann–Whitney U-test or Fisher’s exact test. Statistical significance was accepted as *p* < 0.05 and marked by an asterisk.

### Association between LPLs-producing enzymes and the disease activity

**Figure 1:**
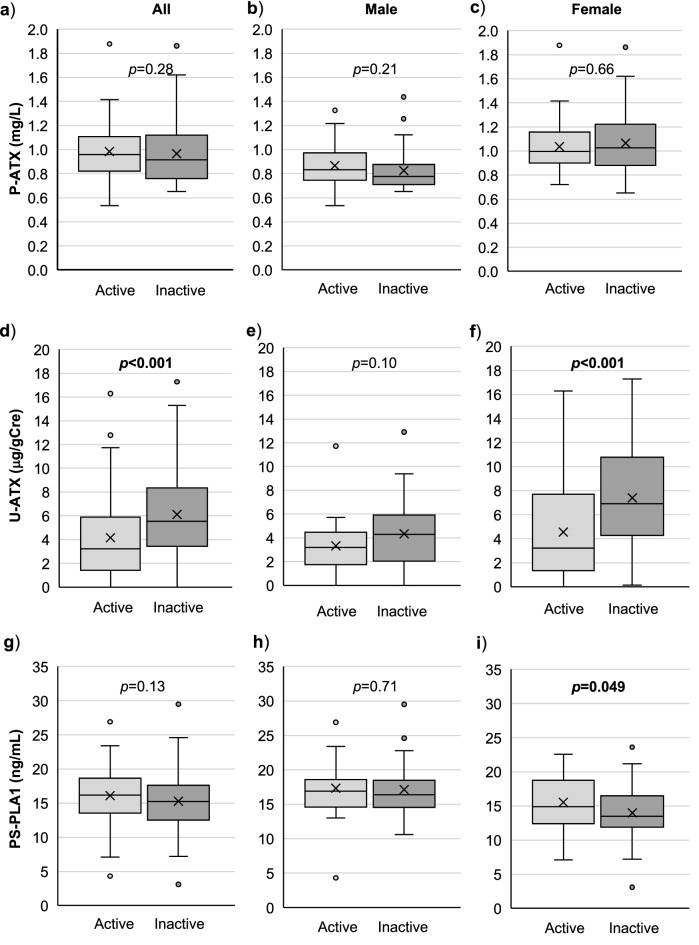
Comparison of the values of LPLs-producing enzymes between patients with active state and inactive state. Comparison of box plot of P-ATX (**a**,**b**,**c**), U-ATX (**d**,**e**,**f**), and PS-PLA1 (**g**,**h**,**i)** levels between active and inactive state. Boxes indicate median and interquartile range, and whiskers indicate minimum–maximum range. Data were analyzed with Mann–Whitney U-test. Statistical significance was accepted as *p* < 0.05 and bold denotes values *p* < 0.05.

We first analyzed the association between LPLs-producing enzymes and the disease activity. As shown in Fig. 1a,g, neither the P-ATX nor PS-PLA1 levels showed significant differences between active and inactive states in all subjects (P-ATX: 0.96 [0.82–1.11] for active state and 0.91 [0.76–1.12] mg/L for inactive state, *p* = 0.28, PS-PLA1: 16.2 [13.5–18.8] for active state and 15.25 [12.4–17.7] ng/mL for inactive state, *p* = 0.13). On the other hand, subjects in an inactive state showed a significant increase in urine-ATX (U-ATX) (3.21 [1.34–6.15] for active state and 5.53 [3.41–8.57] $\upmu$g/gCre for inactive state, *p* < 0.001) (Fig. 1d).

It is known that the P-ATX levels differ between genders^28^. The present study also showed a significant gender difference in the median values of P-ATX (0.79 [0.71–0.92] for males and 1.02 [0.89–1.20] mg/L for females, *p* < 0.001) and U-ATX (3.69 [1.78–5.31] for males and 5.35 [2.23–8.76] $\upmu$g/gCre for females, *p* = 0.01) (Table 1). Interestingly, the plasma levels of PS-PLA1 showed a reverse gender difference (16.5 [14.6–18.6] for males and 14.8 [12.0–17.6] ng/mL for females, *p* = 0.001) (Table 1). Because of these gender differences, we further compared these values between subjects in active and inactive states for each gender (Fig. 1). As in the case of all subjects, neither males nor females showed any differences between active and inactive states in the P-ATX values (*p* = 0.21 for males and *p* = 0.66 for females) (Fig. 1b,c). Notably, female subjects in an inactive state showed a significant increase in the U-ATX values compared with subjects in an active state (3.22 [1.24–7.75] for active state and 6.90 [4.09–10.8] $\upmu$g/gCre for inactive state, *p* < 0.001), while male subjects did not (3.20 [1.52–4.57] for active state and 4.29 [1.96–5.94] $\upmu$g/gCre for inactive state, *p* = 0.10) (Fig. 1e,f). While the PS-PLA1 levels did not show any difference between active and inactive states in all subjects, female subjects in an active state showed a significant increase in those values compared with subjects in an inactive state (14.9 [12.3–18.9] for active state and 13.5 [11.8–16.7] ng/mL for inactive state, *p* = 0.049) (Fig. 1g,h,i). These findings suggest that the levels of U-ATX and PS-PLA1 are associated with the disease inactivity of sarcoidosis, especially in female subjects. We further investigated the correlation between BMI and the levels of U-ATX or P-ATX, which mainly originate from adipose tissues^31^. As shown in supplemental Figure S1, both the P-ATX and U-ATX levels showed no significant correlation with BMI.

### Association between LPLs-producing enzymes and the clinically used biomarkers for sarcoidosis

**Figure 2:**
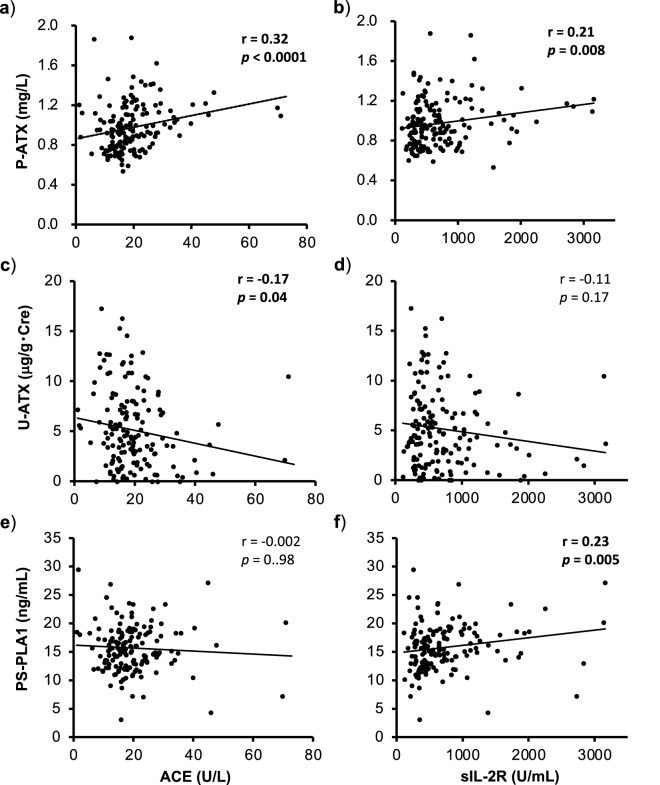
Correlations between LPLs-producing enzymes and clinically used diagnostic biomarkers for sarcoidosis. Correlations between ACE or sIL-2R and P-ATX (**a**,**b**), U-ATX (**c**,**d**) and PS-PLA1 (**e**,**f)**. Data were statistically analyzed by the Spearman’s rank test. Bold denotes values *p* < 0.05.

We next investigated whether these LPLs-producing enzymes are associated with existing clinically used biomarkers such as, serum ACE or serum sIL-2R (Fig. 2). The P-ATX levels showed weak positive correlations with both the ACE levels (r = 0.32, *p* < 0.0001) and sIL-2R levels (r = 0.21, *p* = 0.008) (Fig. 2a,b). The U-ATX levels showed a weakly significant negative correlation with the ACE levels (r = −0.17, *p* = 0.04) (Fig. 2c), but not with sIL-2R levels (r = −0.11, *p* = 0.17) (Fig. 2d). The PS-PLA1 levels showed weak positive correlations with the sIL-2R levels (r = 0.23, *p* = 0.005) (Fig. 2f), but not with the ACE levels (r = −0.002, *p* = 0.98) (Fig. 2e).

We next investigated whether U-ATX is independent of several clinical biomarkers for sarcoidosis (Table 3). Univariate logistic regression analysis with 5 variables revealed that U-ATX, ACE and sIL-2R were associated with the activity of sarcoidosis. After excluding explanatory variables with *p* > 0.05, multivariate logistic regression analysis revealed that U-ATX, ACE and sIL-2R were also independently associated with the activity of sarcoidosis. These findings suggest that the U-ATX levels are independent of the clinically used biomarker of sarcoidosis.

**Table 3:** Univariate and multivariate logistic regression analysis.

	Univariate		Multivariate	
	Odds ratio (95% CI)	p value	Odds ratio (95% CI)	p value
ACE (U/L)	1.13 (1.07–1.20)	** < 0.001**	1.08 (1.01–1.15)	**0.02**
sIL-2R (U/mL)	1.002 (1.001–1.003)	** < 0.001**	1.002 (1.001–1.003)	**0.01**
P-ATX (mg/L)	1.35 (0.36–5.08)	0.66		
U-ATX (mg/g・Cre)	0.87 (0.79–0.95)	**0.001**	0.90 (0.81–0.99)	**0.03**
PS-PLA1 (ng/mL)	1.05 (0.99–1.13)	0.22		

Data were analyzed by univariate and multivariate logistic regression analysis. Bold denotes values *p* < 0.05. Multivariate analysis was performed with ACE, sIL-2R and U-ATX with *p* < 0.05 in univariate analysis. CI: confidence interval.

### Association between U-ATX levels and urine N-acetyl-${\varvec{\upbeta}}$-D-glucosaminidase

**Figure 3:**
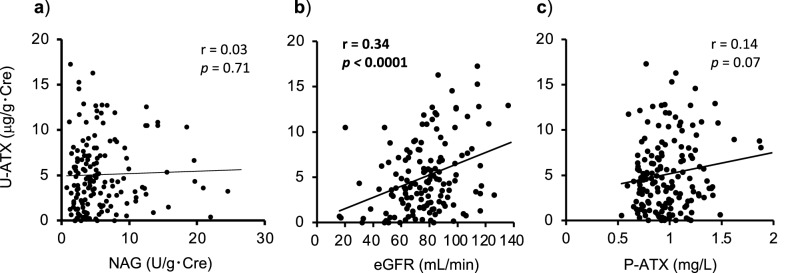
Comparison of the values of the U-ATX levels in relation to the those of the urine NAG, eGFR and P-ATX. Correlations between the values of the U-ATX levels and urine NAG (**a**), eGFR (**b**) and P-ATX (**c**). Data were statistically analyzed by the Spearman’s rank test. Bold denotes values *p* < 0.05.

It has been reported that U-ATX proteins are expressed in the proximal tubular epithelial cells^32^. We further investigated the relationships between the U-ATX levels and the urine N-acetyl-$\upbeta$-D-glucosaminidase (NAG), which is a popular clinical marker for renal tubular injury. The values of urine NAG did not show any correlation to U-ATX (r = 0.03, *p* = 0.71) (Fig. 3a). This finding suggests that the U-ATX levels are independent of the renal tubular injury. In line with a previous report showing that the U-ATX/Cre levels are positively correlated with the estimated glomerular filtration rate (eGFR)^33^, the U-ATX/Cre levels showed a weak positive correlation with the eGFR in this study (r = 0.34, *p* < 0.0001) (Fig. 3b). All subjects with sarcoidosis in this study showed normal eGFR values (Table 1) and the P-ATX levels did not correlate with the U-ATX levels (r = 0.14, *p* = 0.07) (Fig. 3c). These findings suggest that the changes in U-ATX levels are independent of the exudation of P-ATX into urine.

### Association between LPLs-producing enzymes and the transition of disease activity for sarcoidosis

**Figure 4:**
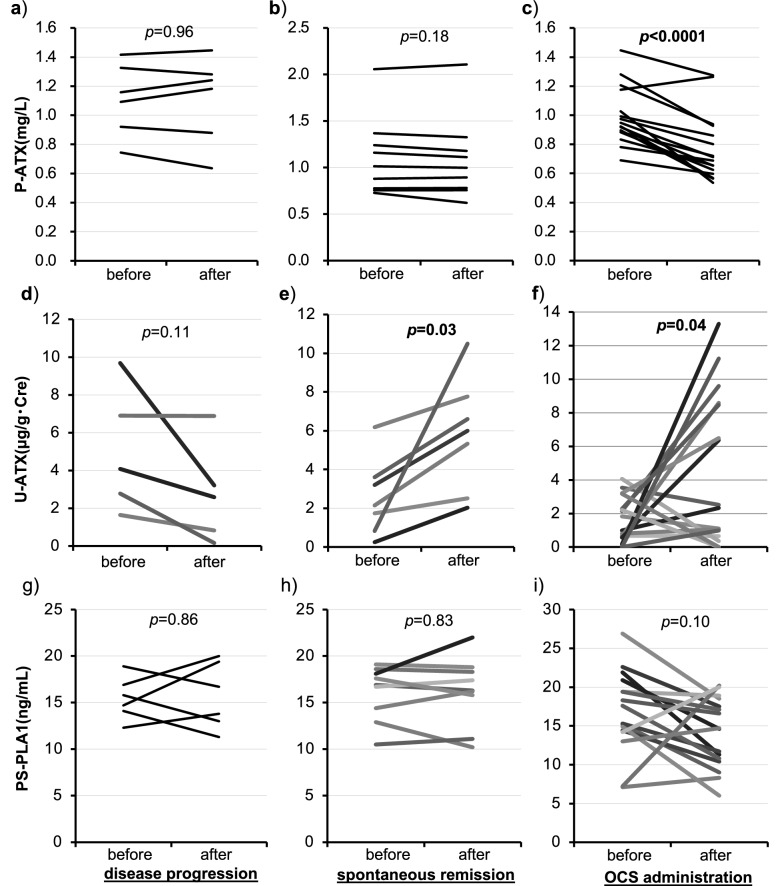
Changes in the LPLs-producing enzymes before and after modification in the clinical status. The comparison of changes in the values of P-ATX (upper row), U-ATX (middle row) and PS-PLA1 (lower row) in paired-samples from the same patients with modification in their clinical status such as disease progression (**a**,**d**,**g**), spontaneous remission (**b**,**e**,**h**) and OCS administration (**c**,**f**,**i**). Data were analyzed by paired t-test. Bold denotes values *p* < 0.05.

During the study periods, we could obtain plasma and/or urine samples repeatedly from 31 subjects every time their disease activities had changed. The clinical characteristics of subjects are summarized in supplemental Table S1. Six paired samples were obtained from sarcoidosis patients with disease progression, nine were from those with spontaneous remission and sixteen were from those with improvement by OCS administration. The associations between LPLs-producing enzymes and the transition of disease activity for sarcoidosis are summarized in Fig. 4.

Neither the P-ATX nor PS-PLA1 levels showed any meaningful change under disease progression and spontaneous remission (Fig. 4a,b,g,h). The U-ATX levels showed a significant increase after spontaneous remission (*p* = 0.03) and tended to decrease after disease progression, though the difference was not statistically significant (*p* = 0.11) (Fig. 4d,e).

After OCS administration, the P-ATX levels showed a significant decrease (*p* < 0.0001), while the PS-PLA1 levels didn’t show any change (Fig. 4c,i). Because OCS reduce the synthesis of ATX, this change in P-ATX would partly reflect the reduction of ATX synthesis, not only the sarcoidosis activity^34^. Notably, the U-ATX levels showed a significant increase even after spontaneous remission (*p* = 0.04), which was similar to the changes after OCS administration (Fig. 4f).

## Discussion

Clinical characteristics and prognosis greatly differ in subjects with sarcoidosis. Although several biomarkers were investigated to predict the disease activity or prognosis, no reliable biomarker has been established^5,35,36^. In the present study, subjects with active sarcoidosis showed a significant decrease in U-ATX levels compared to those with inactive sarcoidosis (Fig. 1d). Additionally, the U-ATX levels tended to decrease after disease progression (Fig. 4d) and increased significantly after the disease activity improved both after spontaneous remission and OCS administration (Fig. 4e,f). These findings suggest U-ATX reflect the disease activity of sarcoidosis. To our knowledge, this is a first report demonstrating a meaningful association between the U-ATX values and the disease activity of sarcoidosis.

The values of U-ATX and PS-PLA1 stratified by the disease activities showed different patterns according to the gender group. The U-ATX levels in whole and female subjects showed significant differences between the active and inactive states, while those in male subjects showed only a non-significant tendency to decrease in the active state. We guess this is because the number of male subjects was relatively smaller than that in females. Another possibility is that the clinical features of sarcoidosis are known to differ according to gender. For example, in Japan, female subjects have a higher incidence than males and biphasic pattern of age-specific incidence^29^. Symptom and organ involvement are also different between males and females^29^. Therefore, we think one cannot exclude the possibility that the gender difference in clinical features of sarcoidosis affects the levels of LPLs-producing enzyme. Further studies with a larger population, especially in male subjects matched in age or other clinical features with females, will be necessary in the future.

The exact origin of U-ATX production has not been determined. A previous report indicated U-ATX might be associated with serum ATX, proteinuria and renal tubular injury^33^. However, we could not find any association between the P-ATX and U-ATX levels (Fig. 3c). In line with our data, it was also reported that it is difficult for the ATX molecule in blood to pass through the normal glomerular filtration barrier due to its high molecular weight^13,37^, although only patients with severe proteinuria (> 3.5 g/gCre) showed an association with the U-ATX concentration^33^. In the present study, most subjects did not have severe glomerular injury and U-ATX showed a weak positive correlation with eGFR (Tables 1, 2 and Fig. 3b). Taken together, it was thought that the U-ATX levels were independent of the P-ATX levels. On the other hand, it was also reported that the expression of ATX was detected in the renal cortex, specifically the proximal tubular epithelial cells and the podocytes in the glomeruli^32^. However, the values of urine NAG did not show any correlation with U-ATX (Fig. 3a). We cannot identify the exact origin of U-ATX production yet, and further basic studies are necessary to elucidate the origin of U-ATX in sarcoidosis.

The present study revealed that the U-ATX levels were significantly lower in an active state than in an inactive state (Fig. 1d, Table 1), while both the serum ACE and sIL-2R were significantly higher in an active state than in an inactive state. We could not find a significant association between these clinical biomarkers and the U-ATX levels in the multivariate logistic regression analysis (Table 3). Likewise, although the values of KL-6 and the presence of cardiac or eye involvement showed significant differences between active and inactive sarcoidosis, we could not find any relationship between these clinical parameters and the U-ATX levels (Data not shown). It was reported that ATX in the local body fluid is independently regulated by unknown mechanisms^38^. Therefore, we guess that there might be some mediators regulating the U-ATX levels and such mediators might affect systemic organs other than kidneys. Since the underlying mechanisms of U-ATX in the pathophysiology of sarcoidosis and its regulatory factors are unkown, further studies will be necessary.

While only female subjects with inactive sarcoidosis showed a significant decrease in the levels of PS-PLA1, neither P-ATX in all, males and females nor PS-PLA1 in all and males showed an association with disease activity of sarcoidosis (Fig. 1a–c,g–i). Although ATX was found in most biological fluids, such as plasma, urine, bronchoalveolar fluid (BALF), synovial fluid and cerebrospinal fluid, the major source of P-ATX is in adipose tissue^31,39^. Therefore, even if ATX production in localized tissue is changed by granulomatous inflammation, the change might be undetectable. In fact, a previous report showed that the changes in the ATX levels in synovial or cerebrospinal fluid are not accompanied by changes in the serum^38^, and suggested that the ATX in urine could be regulated in an independent manner. In line with this speculation, we revealed that both the plasma and urine ATX levels were not correlated with BMI, which reflects the volume of adipose tissue in the whole body. In the present study, most participants showed relatively low values of BMI, which were around 20 to 25. Because these lower values of BMI are not well associated with percent body fat^40^, it is still unclear whether the ATX levels are affected by the amount of adipose tissues or not. Both P-ATX and PS-PLA1 had significant but very weak associations with the clinically used biomarkers, ACE and sIL-2R (Fig. 2a,b,f). Because these associations were too weak to determine the clinical significance, further studies investigating the relationships between LPLs-producing enzymes and existing biomarkers are necessary. In mouse models of rheumatoid arthritis, ATX expression in synovial fibroblasts was stimulated by TNF, which is one of the crucial mediators for granuloma formation in sarcoidosis^21^. Therefore, even though the P-ATX levels showed no significant difference between active and inactive sarcoidosis in the present study, we couldn’t exclude the possibility that P-ATX and PS-PLA1 have some relationship with the pathophysiology of sarcoidosis. Further studies will be required to reveal whether the local expression of ATX or PS-PLA1 changes in the affected organ or tissue.

Concerning the measurement of ATX in the body fluids, there are two procedures for the evaluation. One is the quantity of antigen levels (mg/L in plasma and μg/g$\cdot$Cre in urine), the other is the enzymatic activity (mol/mL/min). Since the serum ATX concentration is strongly correlated with the serum lysophospholipase D activity, it would be a more convenient and accurate method for clinical use^28^. The automated immunoassay system used in this study enables us to handle numerous samples simultaneously, and the results are reported very rapidly, within 22 min^28^. Therefore, we measured the ATX levels by the automated immunoassay system subject to the daily clinical practice.

There are several limitations in this study that should be addressed in the future. First, the present study lacks data from healthy controls and we cannot compare the LPLs-producing enzyme levels of heathy subjects to those of patients with sarcoidosis. However, based on a previous report by one of our colleagues, we think that the U-ATX levels in inactive subjects are distributed in almost the same range as those in control subjects when evaluated by completely identical procedures^33^. These findings suggest that the U-ATX levels decrease abnormally in active sarcoidosis compared with those of healthy or inactive sarcoidosis, and that the U-ATX levels would be considered as a novel marker of disease activity of sarcoidosis. Second, the sample size was relatively small and the follow-up period may not have been long enough. Although many patients with sarcoidosis resolve spontaneously for the first several years, a significant proportion of patients experience progressive exacerbations and remissions for a long time. Third, this is a discovery cohort study and a validation cohort is required to confirm the validity of U-ATX as a biomarker. Further prospective studies with larger samples and healthy controls are necessary to predict the activity of sarcoidosis.

## Conclusion

In conclusion, the present study suggested the U-ATX levels as a novel and useful molecule for assessing the disease activity of sarcoidosis in daily practice. The measurement of U-ATX is likely to be helpful for clinical settings, because it is easy, less invasive and repeatable. Further study is required to elucidate the involvement of ATX in the pathophysiology of sarcoidosis and to develop novel treatment strategies for sarcoidosis.

## Methods

### Patient Population

All patients were diagnosed with sarcoidosis according to the American Thoracic Society (ATS)/European Respiratory Society (ERS)/ World Association of Sarcoidosis and Other Granulomatous Disorders (WASOG) statement on sarcoidosis^41^. Patients with a history of recent (within 1 month before study enrolment) or ongoing OCS and/or immunosuppressants therapy were excluded, because these agents may affect the disease activity and OCS is also known to reduce the synthesis of ATX^34^. Likewise, patients with chronic hepatitis were excluded, because the serum ATX level is known to be elevated in patients with hepatic fibrosis^15^. The disease activity of sarcoidosis was evaluated as active if (1) the patient was newly diagnosed with clinical symptoms such as cough, fever, fatigue and joint pain, (2) the asymptomatic patient had worsening organ involvement during the last 3–6 months follow-up or (3) the number of affected organs associated with sarcoidosis increased during the last 3–6 months follow-up. Inactive sarcoidosis was defined as subjects without clinical symptoms, worsening organ involvement or novel affected organs for at least 3 months. These activities were determined by two independent pulmonologists who were blinded to the measurement of the LPLs-producing enzymes. Patients’ characteristics and medical information including age, sex, smoking status, laboratory findings and radiological imaging data were obtained from the patients’ medical chart.

### Study design

This was a single-center, cross-sectional survey that targeted sarcoidosis patients. The ethics committee of Tohoku University Graduate School of Medicine approved this study (approval number, 2014-1-401) and all participants provided informed written consent. In total, 157 outpatients with sarcoidosis in Tohoku University Hospital were consecutively enrolled in this study from October 1, 2014 to December 31, 2018. The study was carried out in accordance with the Declaration of Helsinki.

### Measurement of plasma ATX, urine ATX and plasma PS-PLA1 levels

Both plasma and urine samples were obtained from patients with sarcoidosis simultaneously and were immediately stored at −80 °C until use. The samples were collected twice or more from some patients whose disease activity had changed spontaneously or by OCS administration. The ATX and PS-PLA1 antigen levels were measured using specific 2-site enzyme immunoassay with TOSOH AIA system (TOSOH, Tokyo, Japan)^28,33,42,43^. Briefly, primary antibody coated with magnetic beads were placed in the reaction cup and alkaline phosphatase-labeled secondary antibody in assay buffer was added to the reaction cup. The ATX and PS-PLA1 assay reagent was prepared by a freeze-dry procedure of the reaction cap. All procedures, such as samples dispensation, incubation of reaction cap, washing and fluorometric detection, were automatically performed by an automated immunoassay analyzer (TOSOH AIA system). Because the ATX antigen levels in urine were lower than the lower limit of the standard ATX measurement, the U-ATX levels were measured using a slightly modified reagent containing a more sensitive anti-ATX monoclonal antibody as compared to the reagent used in the standard assay. The details and performances of the high-sensitive ATX measurement have been described by us previously^33^. On the other hand, the urinary PS-PLA1 levels were not measured because PS-PLA1 in the urine sample was not detectable in the preliminary data.

### Statistical analysis

Data of age, ACE, sIL-2R, KL-6 and pulmonary function were expressed as median and interquartile range (IQR, [Q1-Q3]). The clinical characteristics were compared using Mann–Whitney U-test or Fisher’s exact test. Using a Spearman’s rank test, univariate correlations analyses were assessed, and multivariate logistic regression analyses were performed to investigate the relationship between several biomarkers and LPLs-producing enzymes. Statistical significance was accepted as *p* < 0.05. All statistical analyses were performed using JMP Pro 14 software (SAS Institute, Inc, Cary, NC, USA).

## Supplementary Information

Supplementary Information.

## Data Availability

The datasets generated during and/or analysed during the present study are available from the corresponding author on reasonable request.
