# Supplementary material for: Urine autotaxin levels reflect the disease activity of sarcoidosis
Source: Sci Rep. 2022 Mar 14;12:4372. doi: 10.1038/s41598-022-08388-6 (PMC8921313; doi:10.1038/s41598-022-08388-6)
Supplement: Supplementary file 1 — Supplementary Information. [file 41598_2022_8388_MOESM1_ESM.pdf]

Title

Urine autotaxin levels reflect the disease activity of sarcoidosis

Koji Murakami[1], Tsutomu Tamada[1], Daisuke Saigusa[2], Eisaku Miyauchi[1], Masayuki Nara[3], Masakazu Ichinose[4], Makoto Kurano[5], Yutaka Yatomi[5], and Hisatoshi Sugiura[1]

1 Department of Respiratory Medicine, Tohoku University Graduate School of Medicine, Sendai, Japan

2 Laboratory of Biomedical and Analytical Sciences, Faculty of Pharma-Science, Teikyo University, Tokyo, Japan.

3 National Hospital Organization Akita National Hospital, Yurihonjo, Japan.

4 Osaki Citizen Hospital, Osaki, Japan.

5 Department of Clinical Laboratory, Tokyo University Graduate School of Medicine, Tokyo, Japan

Send correspondence to:

Koji Murakami, M.D., Ph.D.

Department of Respiratory Medicine,

Tohoku University Graduate School of Medicine

1-1 Seiryo-machi, Aoba-ku, Sendai 980-8574, JAPAN

Tel: +81-22-717-8539

Fax: +81-22-717-8549

E-mail: k-mura@rm.med.tohoku.ac.jp

Supplemental Table S1

Characteristics of sarcoidosis patients re-evaluated LPLs-producing enzymes under three conditions

	Disease progression	Spontaneous remission	OCS administration
Number	6	9	16
Gender (Female), n(%)	5 (83%)	7 (78%)	9 (56%)
Age （years）	52 (43-65)	54 (45-61)	48 (36-63)
Active state, n(%)	4 (67%)	8 (89%)	16 (100%)
Sarcoidosis			
Stage0, n(%)	0 (0%)	0 (0%)	0 (0%)
Stage1, n(%)	2 (33%)	3 (33%)	4 (25%)
Stage2, n(%)	4 (67%)	4 (44%)	12 (75%)
Stage3&4 n(%)	0 (0%)	2 (22%)	0 (0%)
Organ involvement			
Eye, n(%)	3 (50%)	5 (56%)	11 (69%)
Skin, n(%)	2 (33%)	1 (11%)	5 (31%)
Heart, n(%)	1 (17%)	2 (22%)	5 (31%)
Others, n(%)	4 (67%)	5 (56%)	11 (69%)
ACE (U/L)	20.6 (14.6-27.2)	23.8 (20.2-36.2)	23.2 (14.0-27.3)
sIL-2R (U/mL)	720 (503-1890)	1020 (545-1915)	846 (700-1530)
P-ATX (mg/L)	1.13 (0.88-1.38)	0.95 (0.76-1.14)	0.94 (0.88-1.14)
U-ATX (μg/g・Cre)	4.08 (2.21-8.29)	2.15 (0.82-3.60)	1.40 (0.58-2.95)
PS-PLA1 (ng/mL)	15.3 (13.7-17.4)	16.9 (13.7-18.4)	16.5 (14.3-20.5)
Follow-up duration (months)	7.5 (5.5-12.3)	10 (6-22.5)	5.5 (2.3-10)

Data are expressed as median and interquartile range (IQR, Q1-Q3). The clinical characteristics were compared using Mann-Whitney U-test or Fisher’s exact test. Statistical significance was accepted as $p < 0.05$ and marked by an asterisk.

Supplemental Figure S1

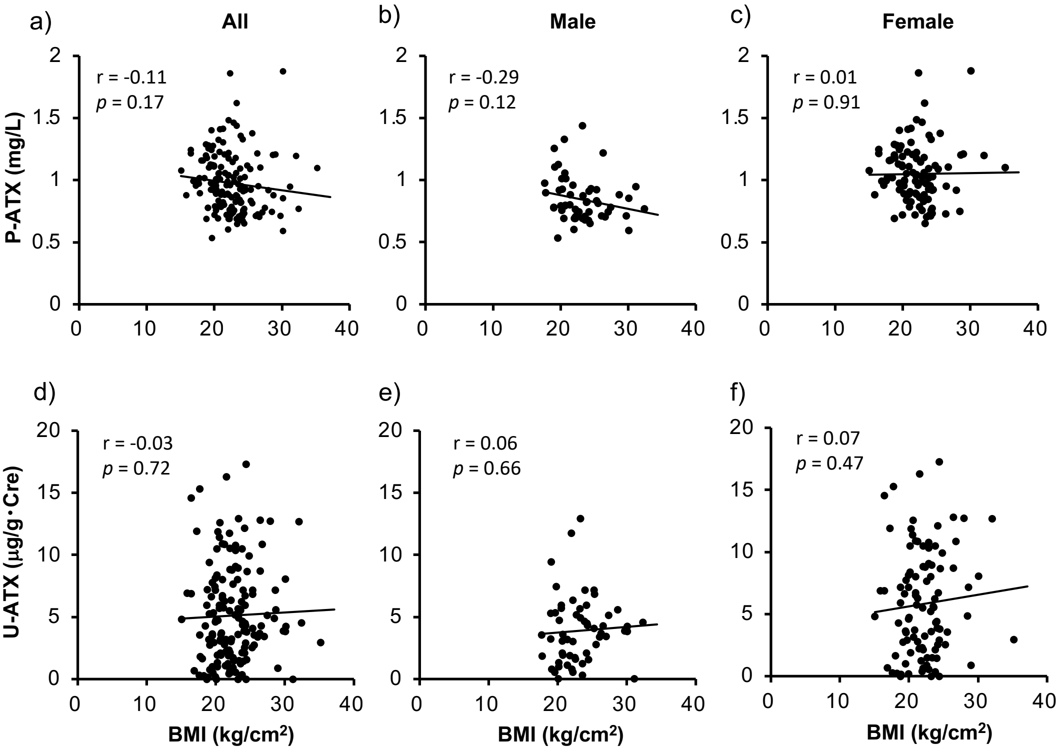

Supplemental Figure S1. Correlations between the values of ATX and BMI.
Correlations between P-ATX (a-c) or U-ATX (d-f) and BMI. Data were statistically analyzed by the Spearman's rank test. Bold denotes values $p < 0.05$.
